# An active human role is essential in big data-led decisions and data-intensive science

**DOI:** 10.12688/f1000research.73876.1

**Published:** 2021-11-08

**Authors:** Mohamed L. Seghier

**Affiliations:** 1 Department of Biomedical Engineering, Khalifa University of Science and Technology, Abu Dhabi, United Arab Emirates

**Keywords:** Knowledge creation, big data, analytics, complexity, data-based decisions

## Abstract

Big data is transforming many sectors, with far-reaching consequences to how decisions are made and how knowledge is produced and shared. In the current move toward more data-led decisions and data-intensive science, we aim here to examine three issues that are changing the way data are read and used. First, there is a shift toward paradigms that involve a large amount of data. In such paradigms, the creation of complex data-led models becomes tractable and appealing to generate predictions and explanations. This necessitates for instance a rethinking of Occam's razor principle in the context of knowledge discovery. Second, there is a growing erosion of the human role in decision making and knowledge discovery processes. Human users’ involvement is decreasing at an alarming rate, with no say on how to read, process, and summarize data. This makes legal responsibility and accountability hard to define. Third, thanks to its increasing popularity, big data is gaining a seductive allure, where volume and complexity of big data can de facto confer more persuasion and significance to knowledge or decisions that result from big-data-based processes. These issues call for an active human role by creating opportunities to incorporate, in the most unbiased way, human expertise and prior knowledge in decision making and knowledge production. This also requires putting in place robust monitoring and appraisal mechanisms to ensure that relevant data is answering the right questions. As the proliferation of data continues to grow, we need to rethink the way we interact with data to serve human needs.

## Introduction

**Figure 1.**
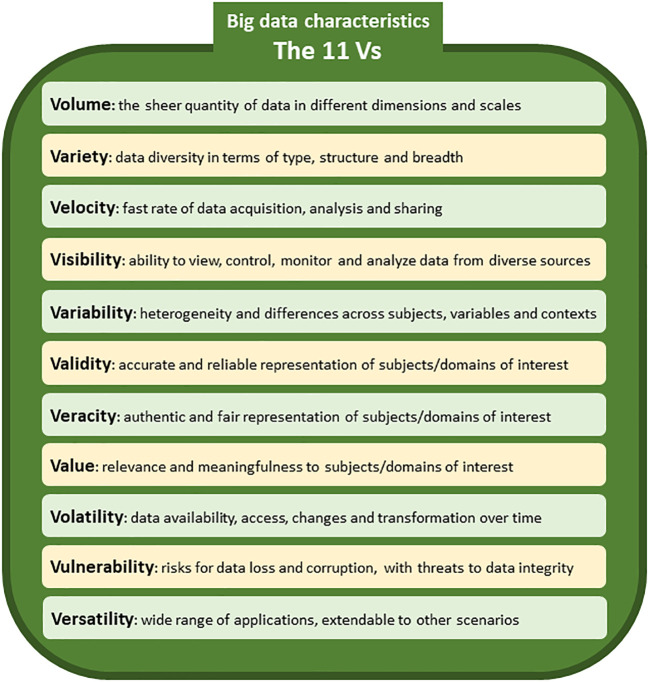
The main characteristics of big data (the 11 Vs).

Big data describes voluminous data that are measured at different pace, scale, size and type. Big data can be produced anytime, anywhere and can come in structured, semi-structured or unstructured formats. Figure 1 illustrates the main characteristics of big data (i.e. the 11 Vs) that make big data challenging and valuable at the same time, ^1^^,^^2^ including volume, variety, velocity, visibility, variability, validity, veracity, value, volatility, vulnerability and versatility, though not all forms of big data possess all eleven characteristics. ^3^ Many sectors have been transformed by the possibility to make accurate and useful decisions by harnessing big data through diverse effective strategies. ^4^ For instance, in the health sector, sophisticated big data analytics can assist doctors to generate accurate and clinically useful individualized predictions for diagnostic or prognostic purposes, which ultimately translate into improved treatments, and more efficient and cost saving service delivery. ^5^^,^^6^ In the education sector, the ability to process big data about students in real-time helps to understand and enhance learner’s behavior and performance and to integrate data into the curriculum. ^7^^,^^8^ As many sectors are competing to take full advantage of the big data revolution, with the potential to generate data-driven ingenious solutions to hard questions, digitization pace is increasing at faster rates thanks to the internet revolution (Internet of Things, 5G) and the widespread adoption of wearable smart devices. Many experts and initiatives around the world consider big data availability as a key driver to transform health and education sectors in the next decades (e.g. ^9^^–^^13^), with far-reaching consequences to how knowledge is produced and shared.

**Figure 2.**
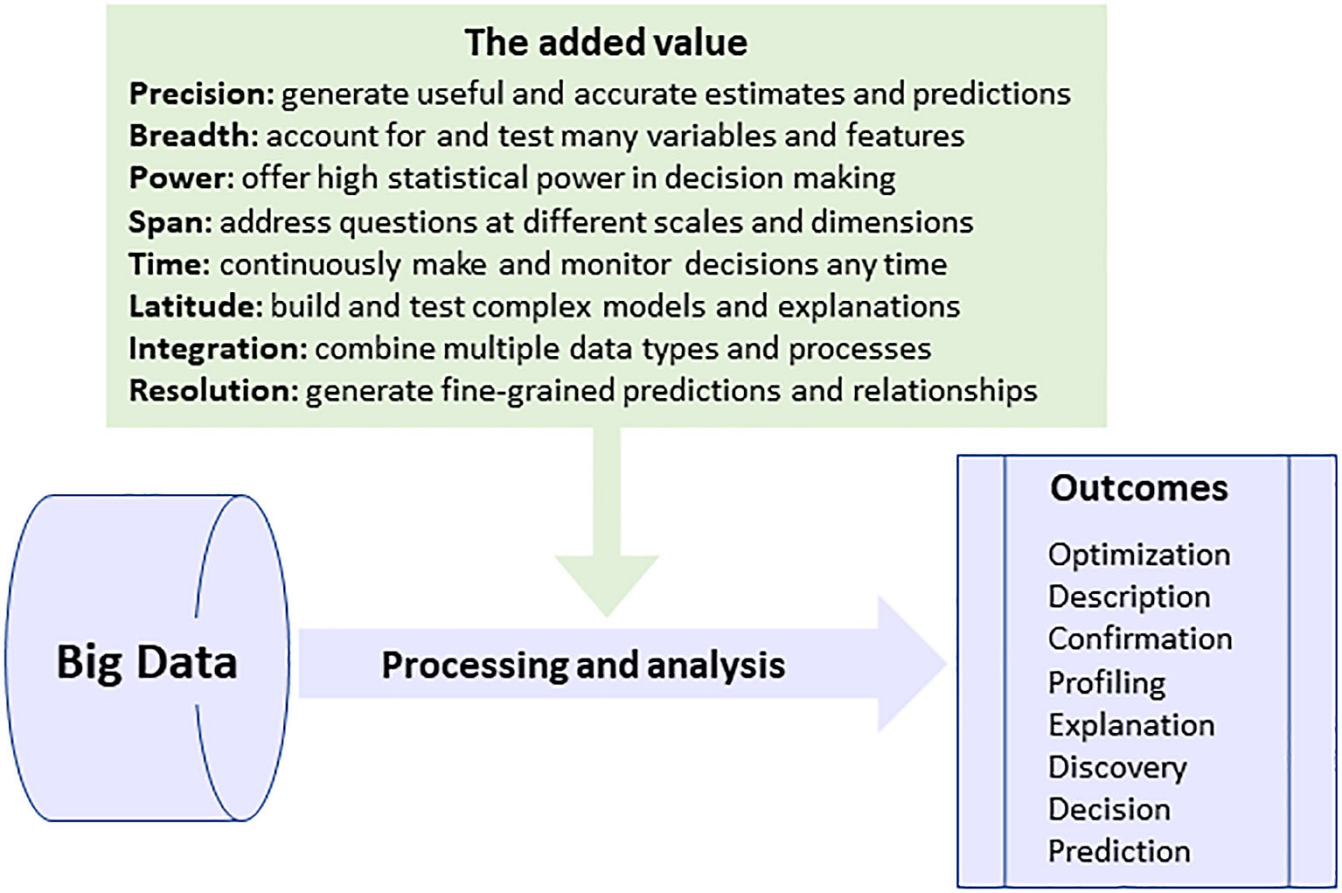
The main added features, based on big data, to knowledge creation and decision-making processes.

For data specialists and policy makers, big data can help tackle problems in a reliable, accurate, unbiased, fast, and comprehensive way. Big data is adding many features to knowledge creation and decision-making processes ( Figure 2). This ‘added value’ is completely reshaping and expanding these processes with the ability to: (1) affect events and systems while they are still unfolding, thanks to the possibility to make fast decisions based on a continuous flow of data; (2) generate an exhaustive and fine-grained picture about every aspect of a student’s learning journey or a patient’s condition in order to create a holistic decision making process; (3) discover patterns and relationships without a priori knowledge or hypotheses, hence minimizing human bias and framing; (4) account for as many variables and features as possible to generate reliable and useful decisions; (5) combine data with different types and from multiple modalities, including unstructured data, to continuously improve the accuracy of decisions; (6) increase mobility where decision making processes can take place anywhere, in the cloud, and on any device; and (7) maximize data sharing and collaboration between different decision makers with the possibility to combine data across diverse sectors and domains to generate in-depth and wide-ranging insights about people and events. These abilities are some of the most frequent arguments listed by decision makers for relying on big data to address a variety of questions in different sectors. ^14^^,^^15^

As the case for any technology, big data does not come free of challenges and limitations. Many challenges have been discussed extensively in the current literature regarding data capture, storage, searching, sharing, integration, transformation, analysis, visualization, consistency, completeness, scalability, timeliness, privacy, security, liability, accountability, governance and ownership. ^8^^,^^16^^,^^17^ The aim of this article is to highlight another side of this big data revolution that warrants further discussion. This concerns the following issues: (1) the transformation of traditional research paradigms with emphasis on volume and quantity, (2) the erosion of the human role in decision making and knowledge discovery processes, and (3) the alluring nature of big data-based decisions. Below we discuss the implication of these issues on how big data is currently sought and treated.

## Big data and good data

Rapid development of processor power and computer parallelization has now made it possible to study huge amounts of data with relative ease. The push for more data is not new as classic models of knowledge discovery emphasize the need for high statistical power, a measure that is positively associated with data size. ^18^ As current analytics and machine approaches require copious amounts of training data, the increase in size and complexity of models’ architecture will make the need for more data even bigger. Under the assumption that machines will be more accurate in extracting relevant and useful information with more and more data, quantity or volume is becoming the dominant feature in big data with sometimes little attention to data quality or relevance; for discussion see. ^19^^–^^21^ Previous work has shown that data volume is not always a significant factor to drive innovation or improve performance. ^22^^,^^23^ This is because the value of a data-driven decision cannot be better than the quality of data that is feeding the machines making that decision. ^24^ Poor or inaccurate decisions can be due to lack of data and/or lack of good data. ^25^ If a model or a process is fed poorly, data-driven features will be poor, and this will only yield poor decisions. ^26^

In this context, as the increasingly attractive solution to understand complex phenomena is to acquire more and more data, traditional inquiry research methods with well-controlled designs and well-selected samples may lose their appeal to researchers, funding agencies and policy makers. ^27^ It is important that collection of good data, even with a limited number of variables from samples that are not large, should not be deemed as a relic from the past. Many discoveries and breakthroughs made in the past, well before this era of big data, were the result of top-quality research with top-quality data that helped to generate accurate mechanistic accounts of many phenomena, mechanistic accounts that human users were able to comprehend, model and harness for diverse applications. ^28^ However, what we learned from many solutions derived from big data is that the processes that yielded a solution remain opaque to human users as the solution itself is sometimes an intrinsic emerging property of big data. This gives very limited insights into the optimal mechanisms that human users can learn and follow to conquer similar problems. A new framework is needed to make sure that solutions derived from big data speak a similar language as solutions derived from prior knowledge by traditional ways of scientific inquiry. ^15^^,^^29^

Another important point concerns the creation of useful models, whether in educational or clinical settings, which is an important endeavor in science. It is apparent that availability of big data will have major ramifications on how models are created and compared. For instance, the design of complex models becomes more appealing as data is getting bigger. Although simple useful models are conventionally preferred (i.e. Occam's razor principle), complex data-led models may shift the way we look for explanations and predictions. Big data can extend the model space to search and assess more complex models with more variables and more features. The aphorism ‘simple is beautiful’ is gradually being replaced by ‘complex is powerful’. Nevertheless, what we notice is that, while data is expanding in volume, this increase in model complexity is not necessarily translating into higher explicability or relevance, i.e. explained variation. Put another way, for a given phenomenon, the proportion of explained variance, as a proxy for the model’s ability to explain that phenomenon, is not growing dramatically with the increase in data volume. This calls for a different way of designing and comparing models given data, as big data are not only answering questions but also generating more.

## Big data with a human touch

The way big data is growing in its complexity, volume or type, makes it a product not crafted for human use or comprehension. How big data is typically created and collected illustrates that its purpose is mainly to feed sophisticated analytics, with little concern to how human users will read data. ^30^ The human aspect is gradually left aside in the processing of big data and in the making of big data-driven decisions, hence reducing human contribution to merely conveying the output of data processing tools. What’s more concerning is that human users’ ability to interpret and make sense of such big data-driven decisions is continuously eroded, with sometimes no say on how such analytics should read, process, summarize and present big data. When big data is submitted to an AI-powered tool for instance, the way it is cleaned, reduced and processed bear no attempt to account for what is relevant for the human end-users. The education sector provides an interesting example about the implications of this issue. In this sector, there is a growing interest in using big data and analytics to predict students’ performance for diverse applications (for a recent systematic review, see ^31^), with the ultimate goal to personalize learning and provide adequate and timely academic support. ^8^^,^^32^ However, when students for instance are denied enrolment in a program or a track because an AI-powered warning system decided that they have an above-threshold risk of failing courses, this high-stake decision needs careful examination and opportunity for re-evaluation for a better protection of students’ rights. ^33^ There is an even increasing risk that decision makers will hide behind the sophistication of such AI-powered tools, leaving no opportunity to challenge or question decisions because they have been made by intelligent machines on massive data, with the assumption that such decisions are, by design, error-free and unbiased. It is thus vital that opportunities for human involvement are encompassed in big data collection and processing. Processes that value the human input on top of big data-driven features should be supported. ^30^^,^^34^ A proper dialogue needs to take place between policy makers and developers to create processes and tools that are accessible to all and that are in the service of the human decision makers.

We need big data with a human touch. Ongoing advances will increase big data’s dual potential to either empower or isolate human users from decision making and knowledge creation processes. It is thus vital to gradually build synergies that can bring big data to their utmost purpose which is to serve human needs. Policy makers should put in place safeguards and regulations to monitor how big data are collected and treated to address a given question. Transparency should be guaranteed at all levels, in particular regarding the exact purpose of collected data and how it is going to be manipulated and processed. ^35^ When big data-driven decisions are made, the concept of legal responsibility and liability may become fuzzy. As analytics and machine learning algorithms are becoming increasingly impenetrable, decision makers are not always able to fully comprehend the breadth and consequences of a given data-driven decision. Hence, accountability needs to be clarified when wrong or inappropriate decisions or predictions are made with big data. ^36^ It is important to be able to trace back all processes and stages involved in the making of a decision. There must be integrated tools that analyze and interrogate the making process of a decision in case of an inquiry or an appeal. ^37^ Perhaps most importantly, the decision-making process must be freed from the opacity of big data and analysis methods, for example by scrutinizing the type of data selected or omitted in the process, the underlying assumptions behind the selected mathematical models, and the anticipated consequences of the decisions in the light of what is socially and legally acceptable. ^17^^,^^38^^,^^39^

## The seductive allure of big data

When a presumably persuasive but irrelevant information is added to an argument, naïve or nonexpert users might be influenced by that information. For example, nonexperts may judge satisfying some bad explanations that are supported by irrelevant information that has a persuasive power such as a neuroscience evidence or brain images. ^40^^,^^41^ What this suggests is that, when people are nonexperts or cannot comprehend a process, they tend to be influenced (seduced) by other irrelevant peripheral features ^42^; for example, to be persuaded by peripheral aspects like an image of a brain, a complex equation, a massive number, or a photo of a famous person. Big data is no exception; nonexpert users and decision makers might be seduced by its size and complexity. Hence, there is a risk that decisions become more persuasive and significant just because the process used to generate them is based on big data. Decision makers might be tempted to give more authority (evidence) to decisions or information generated from big data. This might undermine appeals and grievance procedures if decisions and solutions are portrayed as the output of infallible machines on voluminous data.

It is thus important to dissociate core processes in big data-based decision making from peripheral aspects of that process. The way big data is visualized, presented and portrayed should not be made with the desire to obfuscate or complicate the information conveyed to users. When nonexperts are overwhelmed by the sheer complexity and the degree of details that result from a decision-making process on big data, they tend not to question such process. Data specialists should therefore devise user-friendly ways to make core processes as clear and as coherent as possible, such as how data is cleaned, reduced and what relevant features are selected. Additional data quality assurance processes must be put in place to ensure that big data is serving its main purpose in the most accurate and valid ways, using data sampling and profiling to minimize the risk for data quality degradation ^43^ and systematic processes to ensure data validity for other purposes (i.e. repurposed data). ^44^

This seductive nature of big data has implication on ethics. Results that emerge in a data-driven manner might not always be appropriate from an ethical point of view (e.g. results that stigmatize a group by gender, ethnicity or health condition), and participants from different groups might feel their data are being used or represented in ways they cannot fathom. This can create a divide between those who access and control big data and those who merely feature in them. ^45^^,^^46^ Big data collection can be more invasive than necessary due to easy access, ^47^ and it is not unusual to see big data used to answer different questions than the ones originally consented for by participants. ^48^ Big data often contains much more information than is strictly necessary, hence guidelines and safeguards must be put in place to ensure fairness, equity and transparency. It is important that policy makers and ethics bodies uphold the same standards to big data given its longevity and intrusive nature. It is too easy to impress with big numbers, and it is a moral and legal obligation to communicate the right results with their very real limitations to society and policy makers. ^49^

## Conclusion

Big data holds the potential for revolutionizing many sectors. We have seen the birth of many big data initiatives, ^50^ for instance UN's Global Pulse initiative, Europe’s Data Saves Lives initiative, NIH’s All of Us program, China’s Cohort Consortium, and the UK Biobank. But big data is not the answer to all questions, regardless of how attractive and impressive big data is. As machines can process big data and discover new relationships between variables of any arbitrary shape, it is important to appraise the whole process by making sure that big data are answering the right questions. As we are moving toward a more data-intensive type of science, the objective must remain to understand than merely predict relationships. This necessitates a hybrid framework that incorporates insights from human experts in the data-led knowledge production. We are beholding how big data is gaining unprecedented authority in the decision making and knowledge production processes, while present standards (methodological, ethical and legal) are not able to keep pace with current growth in big data and their ramifications for many sectors. There is an urgent need for more critical reflection on how humans should interact with data and data-driven information.

## Data availability

No data is associated with this article.
