# Supplementary material for: An active human role is essential in big data-led decisions and data-intensive science
Source: F1000Res. 2021 Nov 8;10:1127. [Version 1] doi: 10.12688/f1000research.73876.1 (PMC10905148; doi:10.12688/f1000research.73876.1)
Supplement: Peer review history

# Reviewer report: Reviewer response for version 1

**DOI:** 10.5256/f1000research.77562.r238781

**Published:** 2024-02-28

**Authors:** Tariq Rahim Soomro

**Affiliations:** 1 Institute of Business Management, Karachi City, Pakistan

The article discusses the transformative impact of big data on various sectors, highlighting its potential benefits and challenges. It focuses on three key issues: the transformation of traditional research paradigms, the diminishing human role in decision-making processes, and the seductive nature of big data. 

**Strengths:**

1. The article provides a comprehensive overview of the characteristics of big data, its applications across different sectors, and the challenges associated with its utilization.
2. The article critically examines the prevailing trend of emphasizing volume over data quality. It rightly emphasizes that the value of data-driven decisions is contingent on the quality of the data.
3. The discussion on the erosion of the human role in decision-making processes is thought-provoking. It underscores the importance of considering human interpretation and involvement in big data-driven decisions, especially in critical areas like education.
4. The article appropriately highlights ethical considerations associated with big data, emphasizing the potential biases and the need for transparency, accountability, and safeguards in data collection and processing.

**Areas for Improvement:**

1. The article could benefit from clearer transitions between its main points. At times, the flow of arguments might be challenging to follow, affecting the overall coherence of the text.
2. While the article mentions instances in healthcare and education where big data can be advantageous, providing more concrete examples and case studies could enhance the reader's understanding of these applications.
3. While the article outlines challenges, it could provide a more balanced discussion by also highlighting successful implementations of big data and ways in which these challenges are being addressed in current practices.
4. The article references initiatives and programs up to November 2023, and considering the rapid evolution of technology and data-related fields, more recent references could strengthen the arguments.
5. Author may avoid using the terms, such as "we", "our", etc.
6. The Author may consider the following relevant research articles: Naqvi *et al.* (2021 ^1^); Tariq (2015 ^2^).

**Overall:**

 The article presents a valuable critique of the current trends in big data utilization, urging for a balanced approach that considers data quality, human involvement, and ethical considerations. Despite some areas for improvement, the article contributes to the ongoing discourse on responsible and effective big data practices.

Is the topic of the opinion article discussed accurately in the context of the current literature?

Yes

Are arguments sufficiently supported by evidence from the published literature?

Yes

Are all factual statements correct and adequately supported by citations?

Yes

Are the conclusions drawn balanced and justified on the basis of the presented arguments?

Yes

Reviewer Expertise:

Big Data, IoT, GIS, IDNs, Cybersecurity, Quality Education

I confirm that I have read this submission and believe that I have an appropriate level of expertise to confirm that it is of an acceptable scientific standard.

## References

[1] : The nexus between big data and decision-making: A study of big data techniques and technologies. *The International Conference on Artificial Intelligence* .2021; Reference source

[2] : Big Data Challenges. *Computer Engineering & Information Technology* .2015;04(03) : 10.4172/2324-9307.1000133 10.4172/2324-9307.1000133

# Reviewer report: Reviewer response for version 1

**DOI:** 10.5256/f1000research.77562.r126888

**Published:** 2022-03-24

**Authors:** Marta Ewa Kuc-Czarnecka

**Affiliations:** 1 Department of Statistics and Econometrics, Faculty of Management and Economics, Gdańsk University of Technology, Gdańsk, Poland

The article addresses significant problems of the impact of visible numbers (scoring, rankings) and invisible numbers (algorithms) used due to the growing popularity of big data. Important aspects such as overinterpretation of data, blurring of messages, misleading the recipient (intentionally or not), lack of transparency, and too much confidence in the number at the decision-making stage were noted. I believe that reasonable and gentle voices pointing out the problematic issues are vital. Especially in the era of admiration for measuring everything and everywhere, as well as over-modelling and numerification of reality. Perhaps the authors could also devote a bit of space to the issue of big data and fake news - on the one hand, big data should help in capturing them, and on the other hand, they are often used in disinformation or propaganda. I also recommend the authors to read:

- Saltelli, A., Dankel, D., Di Fiore, M., Holland, N. & Pigeon, M. *Science, the Endless Frontier of Regulatory Capture*. https://papers.ssrn.com/abstract=3795058 (2021) doi:10.2139/ssrn.3795058.
- Saltelli, A. *et al.* Five ways to ensure that models serve society: a manifesto. *Nature* **582**, 482–484 (2020).
- Bruno, I., Didier, E. & Prévieux, J. *Statactivisme. Comment lutter avec des nombres*. (Zones, La Découverte, 2014).
- Saltelli, A. *et al.* *Why ethics of quantification is needed now*. *UCL Institute for Innovation and Public Purpose, Working Paper Series* (2021).
- Couldry, N. & Mejias, U. A. Data Colonialism: Rethinking Big Data’s Relation to the Contemporary Subject. *Telev. New Media* **20**, 336–349 (2019).

I have mentioned these articles because they refer to quantification in its broadest sense and its impact on decision-making, as well as the often questionable ethical use of numbers. Perhaps the author, on the basis of these works, will be able to expand the scope of his research in the future.

 I believe that the article in its current form meets the formal and substantive requirements, and therefore is suitable for indexing.

Is the topic of the opinion article discussed accurately in the context of the current literature?

Yes

Are arguments sufficiently supported by evidence from the published literature?

Yes

Are all factual statements correct and adequately supported by citations?

Yes

Are the conclusions drawn balanced and justified on the basis of the presented arguments?

Yes

Reviewer Expertise:

ethics of quantification, big data and ethics, composite indicators, taxonomy, spatial statistics, GIS.

I confirm that I have read this submission and believe that I have an appropriate level of expertise to confirm that it is of an acceptable scientific standard.

## References

[1] : Science, the Endless Frontier of Regulatory Capture. *SSRN Electronic Journal* .2021; 10.2139/ssrn.3795058 10.2139/ssrn.3795058

[2] : Five ways to ensure that models serve society: a manifesto. *Nature* .582(7813) : 10.1038/d41586-020-01812-9 482-484 10.1038/d41586-020-01812-9 32581374

[3] : Comment lutter avec des nombres.

[4] : Why ethics of quantification is needed now. *UCL Institute for Innovation and Public Purpose, Working Paper Series (IIPP WP 2021/05)* . Reference source

[5] : Data Colonialism: Rethinking Big Data’s Relation to the Contemporary Subject. *Television & New Media* .2019;20(4) : 10.1177/1527476418796632 336-349 10.1177/1527476418796632
